# Persistence of SARS-CoV-2 Infection in Severely Immunocompromised Patients With Complete Remission B-Cell Lymphoma and Anti-CD20 Monoclonal Antibody Therapy: A Case Report of Two Cases

**DOI:** 10.3389/fimmu.2022.860891

**Published:** 2022-04-14

**Authors:** Carlos Martínez-Chinchilla, Lucía Vazquez-Montero, Natalia Palazón-Carrión, Isabel M. Fernández-Román, José López-Barba, Luis de la Cruz-Merino, Jesús Rodríguez-Baño, Zaira R. Palacios-Baena

**Affiliations:** ^1^ Hematology and Hemotherapy Unit, University Hospital Virgen Macarena, Seville, Spain; ^2^ Clinical Oncology Department, University Hospital Virgen Macarena, Seville, Spain; ^3^ Department of Medicine, University of Seville, Seville, Spain; ^4^ Infectious Diseases and Microbiology, University Hospital Virgen Macarena, and Biomedicine Institute of Sevilla (IBIS)/CSIC, Seville, Spain; ^5^ Centro de Investigación Biomédica en Red en Enfermedades Infecciosas (CIBERINFEC), Madrid, Spain

**Keywords:** SARS-CoV-2, COVID-19, immunocompromised, anti-CD20, rituximab, lymphoma, case report

## Abstract

Immunosuppressant conditions such as hematological malignancies increase the risk of severe acute respiratory syndrome coronavirus 2 (SARS-CoV-2) infection. It has been described in the literature that patients on anti-CD20 maintenance therapies for lymphoid malignancies are susceptible to having recurrent flares together with viral replication or reinfections, although these cases are scarce. These patients are not well represented in randomized controlled trials, and as a consequence, the evidence for the use of certain treatments in this scenario is lacking. We present two cases of patients with B-cell lymphoma on remission and treated with rituximab on maintenance. They developed at least 1 flare of coronavirus disease 2019 (COVID-19) after acute infection and always after receiving rituximab. RT-PCR was positive in the nasopharyngeal swab and also in plasma. Patients were treated during flares with remdesivir, hyperimmune plasma, and corticosteroids. These two cases showed the unresolved problem of COVID-19 in immunosuppressant patients and showed that despite the vast amount of information available on SARS-CoV-2, information in this subgroup of patients is lacking.

## Introduction

In December 2021, more than 5 million people died because of severe acute respiratory syndrome due to severe acute respiratory syndrome coronavirus 2 (SARS-CoV-2) infection^1^. Immunosuppressant conditions such as hematological malignancy or cancer increase the risk of severe infection or death up to 25%–34% in hospitalized patients (1, 2). While viral load in non-immunocompromised patients peaks 5–7 days after initiation of symptoms and declines around 10 days after infection coinciding with IgG seroconversion, the duration of viable shedding in immunocompromised patients is longer and may last for months (3). In addition, these patients may have relapses of viral replication with recurrent severe symptoms (4, 5), which are associated with a lack of adequate immune response to infection or vaccination. Both cellular and humoral immune responses play important roles in viral clearance and associated symptoms. Persons with lymphoid malignancies, especially those with B-cell-depleting therapies such as anti-CD20 drugs are particularly susceptible to having flares and lack of response to vaccination due to impaired humoral immunity and the ability to produce neutralizing antibodies (6–8). These patients are often excluded from studies and urgently need therapeutic options.

We described two cases of relapsed COVID-19 symptoms and viral replication in patients with lymphoma and B-cell-associated immunosuppression. Both patients were aware of the dissemination of this report and provided written consent for this publication.

## Case Report 1

**Figure 1:**
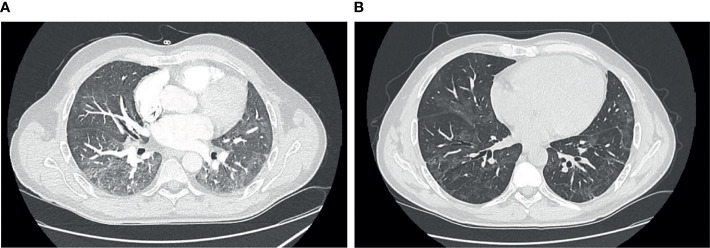
**(A)** Chest computer tomography (CT) at day +75 showing bibasal peripheral ground-glass opacities. **(B)** Chest CT at day +117 showing persistence of ground-glass opacities with significant reduction.

**Figure 2:**
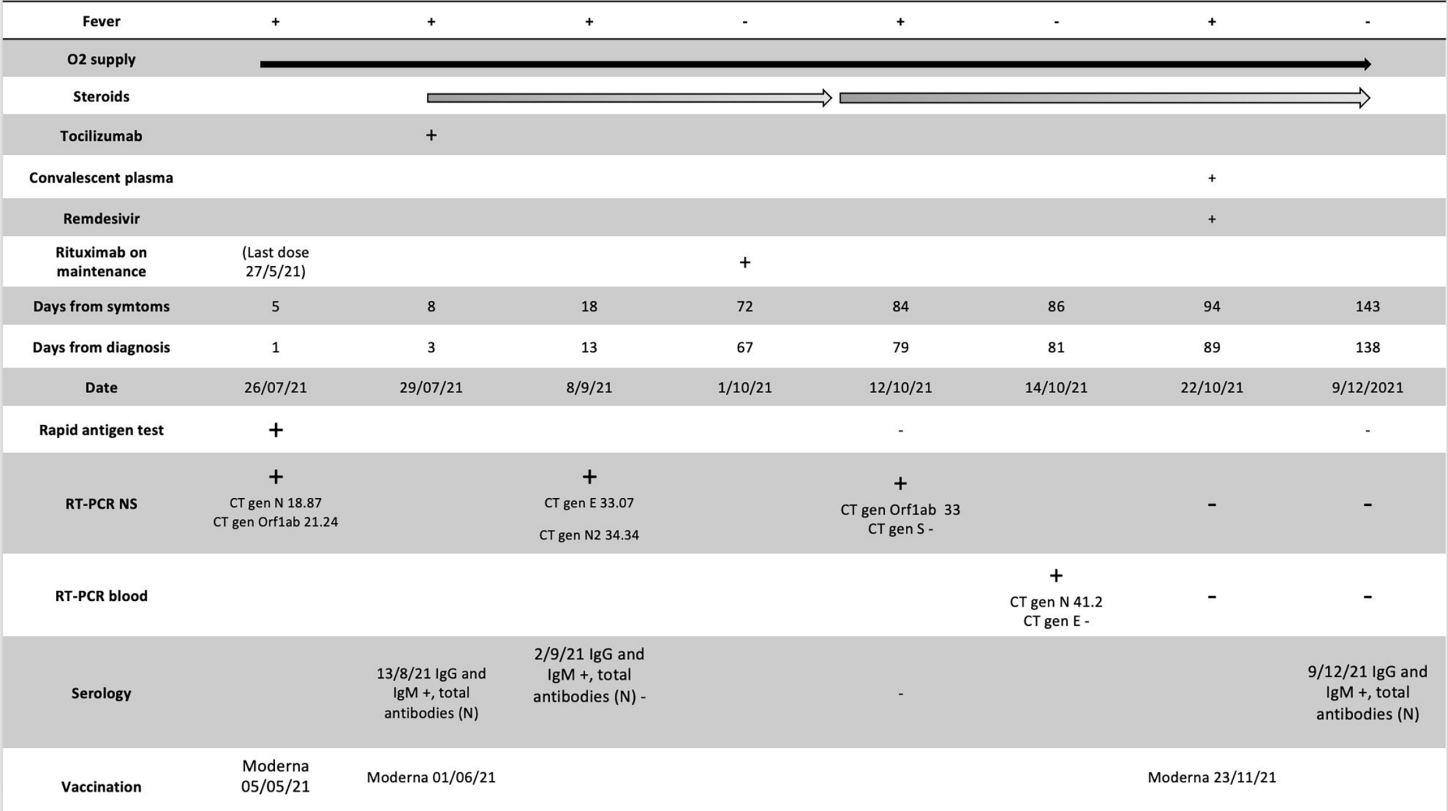
A timeline of the case 1; administered treatments, O2 supply, Rituximab administration, serology, date of vaccination and diagnosis test [Rapid Antigen Test and RT-PCR in nasopharyngeal swabs (NS) and plasma]. RT-PCR, real-time polymerase chain reaction; CT, Cycle threshold values; N, Normal.

A 47-year-old male patient affected by dilated cardiomyopathy associated with filamin C variant started with drenching night sweats and unexplained weight loss. He was diagnosed in March 2020 with stage IV-B follicular non-Hodgkin lymphoma, categorized as high risk (3 points) according to the International Follicular Lymphoma Prognostic Factor Project (FLIPI). In April 2020, he was started chemoimmunotherapy R-CHOP schedule (rituximab 375 mg/m^2^ day 1, doxorubicin 50 mg/m^2^ day 1, vincristine 1.4 mg/m^2^ day 1, cyclophosphamide 750 mg/m^2^ day 1, and prednisolone 100 mg/24 h days 1–5) once every 21 days, completing six cycles. The treatment ended in August 2020, with a partial response as assessed by positron emission tomography (PET-CT) in September 2020 according to Cheson’s criteria. In October 2020, maintenance treatment with rituximab 375 mg/m^2^ bimonthly was started. A follow-up PET-TC in May 2021 after fourth rituximab doses documented lymphoma remission. A fifth dose of rituximab was administered in May 2021; he received 2 doses of the mRNA-1273 vaccine in May and June 2021. In July 2021, he was admitted to the hospital after 5 days of fever and cough; oxygen saturation at room air was 98% without dyspnea. Chest radiograph showed bilateral pneumonia. Blood tests showed the following: moderate neutropenia and thrombocytopenia (710 and 84.000/L, respectively), normal lymphocyte count, elevation of lactate dehydrogenase (613 U/L; cutoff <250 U/L), CRP (89 mg/L; cutoff <5 mg/L), interleukin-6 (79 pg/ml; cutoff <7 pg/mg), and ferritin (539 ng/mg; cutoff <290 ng/mg), and mild IgM hypogammaglobulinemia (37 mg/dl; normal value, 50–300), with normal levels for the other immunoglobulins. A peripheral blood flow cytometry showed a depletion of B lymphocytes (CD19 0%, CD3 85%) with an inversion of the CD4+/CD8+ cell ratio (0.55, cutoff 1–2). A nasopharyngeal swab was positive to SARS-CoV-2 by RT-PCR. After 3 days of admission (+8 since symptoms onset), oxygen saturation dropped to 88%; high-flow nasal oxygen, dexamethasone, and two doses of tocilizumab were administered. On day +10 after the onset of symptoms, the patient was transferred to the ICU, where non-mechanical ventilation was started. After 9 days in the ICU, he was slowly weaned off from oxygen. A nasopharyngeal swab was persistently positive by RT-PCR, detecting the positivity for gene E (cycle threshold (Ct), 33.07) and gene N (Ct, 34.34) with Xpert^®^ SARS-CoV-2 equipment. Serological testing did not detect antibodies against SARS-CoV-2 (neither antinucleocapsid nor antispike antibodies, day +18 from symptoms onset). He was discharged in September 2021 (+62 days from the onset) with home oxygen therapy. In October 2021, he resumed treatment with rituximab (sixth dose). A computer tomography scan (CT) was performed as monitoring of his sequelae after acute and severe COVID-19 and revealed ground-glass opacities in both basal lobes of the lungs (**Figure 1A**) . Eight days after receiving rituximab (+81 days), he was readmitted due to fever and cough. Rapid antigen test of SARS-CoV-2 was negative, and a RT-PCR was performed in nasopharyngeal swab detecting gene ORF1ab gene (Ct 33) but not gene S. A RT-PCR in plasma with detecting gene N2 (Ct 41.2) was also performed. All microbiologic tests for other respiratory pathogens were negative. Due to the hypothesis of persistent replication of SARS-CoV-2 and flare of COVID-19 in an immunocompromised host, hyperimmune plasma was infused (day +91 from the onset of symptoms), and remdesivir was administered for 10 days (200 mg initially continued with 100mg/day). He also received high doses of corticosteroids (1 mg of prednisone per kg with slow tapering); the patient improved and was discharged, remaining afebrile but still needing supplementary oxygen at home. A month later, a new follow-up CT scan was performed (**Figure 1B**), showing a significant improvement of the infiltrates; also, oxygen flow needs were decreasing. In November 2021, he received the third dose of the mRNA-1273 vaccine. In December 2021, SARS-CoV-2 RT-PCR in plasma and nasopharyngeal swab were negative. Serological testing of antibodies was still negative. Now, despite having a previous physically active lifestyle, he is limited by the need for home oxygen. The clinical course, diagnostic tests, and treatments received are summarized in **Figure 2**.

## Case Report 2

**Figure 3:**
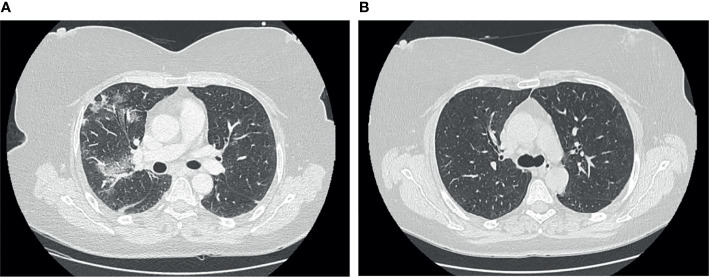
**(A)** Chest computer tomography (CT) at day +34 showing peripheral ground-glass opacities in both basal lobes of the lungs. **(B)** Chest CT at day +83 showing a complete disappearance of ground-glass opacities.

**Figure 4:**
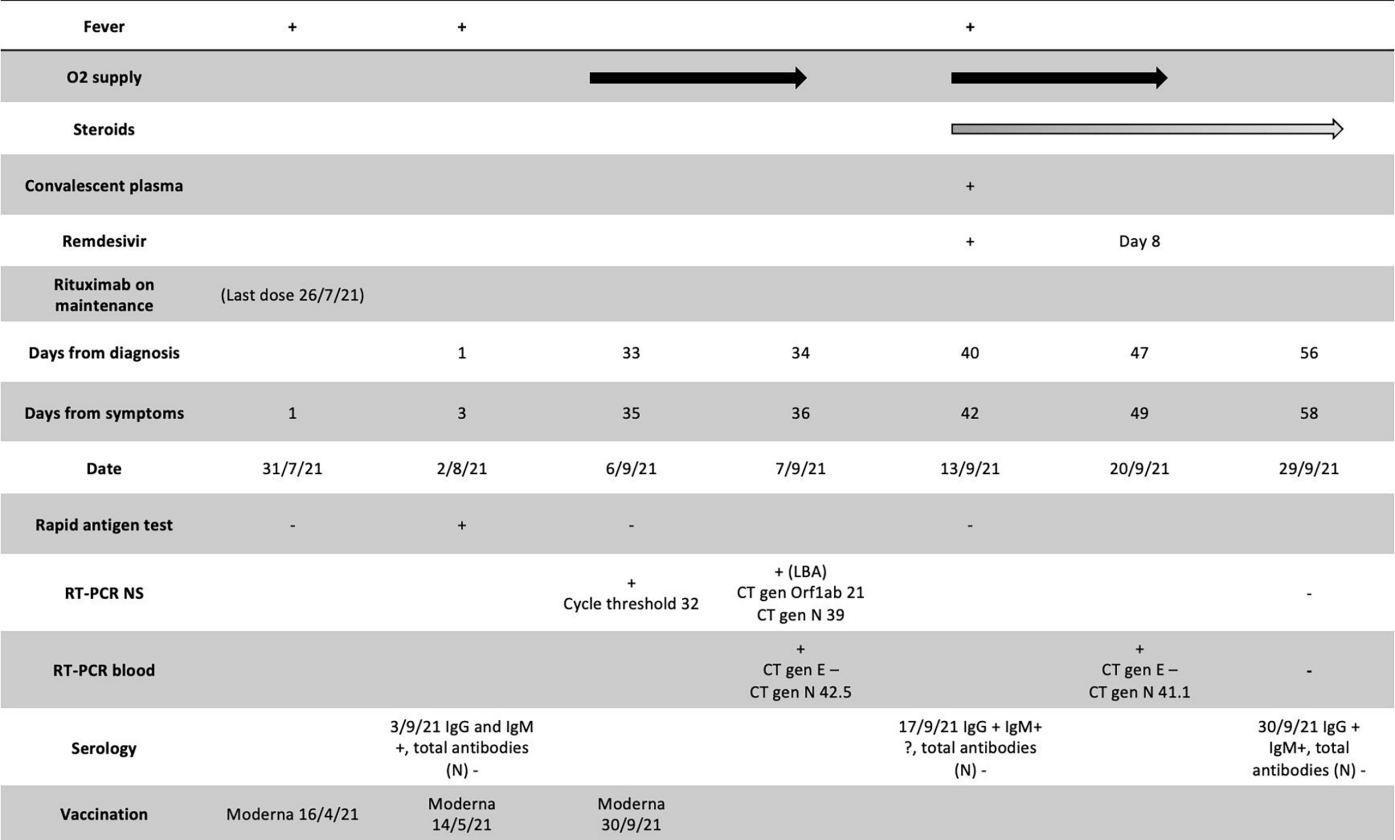
A timeline of the case 2; administered treatments, O2 supply, Rituximab administration, serology , date of vaccination and diagnosis test ( Rapid Antigen Test and RT-PCR in nasopharyngeal swabs (NS) and plasma). RT-PCR, real-time polymerase chain reaction; CT, Cycle threshold values; N, Normal.

A 51-year-old woman with a history of smoking and dyslipidemia was diagnosed with grades 1–2 stage IV follicular non-Hodgkin lymphoma in January 2020, categorized as intermediate risk (2 points) according to the FLIPI index. Similar to the above patient, she received 6 cycles of chemoimmunotherapy R-CHOP schedule until July 2020, with an almost complete metabolic response in PET-CT performed at the end of treatment. Two months later, in October 2020, she began maintenance therapy with rituximab 375 mg/m^2^ in monotherapy every 2 months with an expected duration of 2 years. She was vaccinated with mRNA-1273 (second dose on May 2021). On July 26, 2021, she received the fifth cycle of rituximab. On August 2, 2021, she tested positive for SARS-CoV-2 rapid antigen on a nasopharyngeal swab after 2 days of low-grade fever and malaise, which had started 5 days after the last rituximab dose. For 20 days, the patient was followed up at home and was evaluated at the emergency department twice because of intermittent fever, nausea, occasional vomiting, and dyspnea on moderate exertion. On both occasions, oxygen saturation was normal, chest radiograph showed absence of infiltrates, and blood cultures were negative. On September 1, 2021, on day +30 of illness, she was admitted to the hospital due to persistent symptoms and oxygen saturation on room air at 92%. A new SARS-CoV-2 rapid antigen test on a nasopharyngeal swab was performed with a negative result at admission. Blood tests showed the following: slight elevation of lactate dehydrogenase (263 U/L; cutoff <250), alanine transaminase (83 U/L, upper-level limit 40 U/L), and CRP (40 mg/L; cutoff <5 mg/L), with normal lymphocyte count; SARS-CoV-2 serology showed vaccine-related response but not to natural infection (positive for anti-S IgM and IgG and negative for anti-N antibodies). During admission, a chest and abdominal CT scan was performed, showing bilateral interstitial pneumonia and no images suggestive of lymphoma progression (**Figure 3A**). A new nasopharyngeal swab was positive by RT-PCR, with Ct 32. Bronchoscopy was performed; RT-PCR was positive for SARS-CoV-2 gene N (Ct 39) in bronchoalveolar lavage and negative for other respiratory pathogens. β2-Microglobulin, used as a tumor marker in the follow-up of lymphoproliferative syndromes, was slightly elevated in the blood (2.97 mg/L; cutoff <2.20 mg/L). A peripheral blood flow cytometry showed a depletion of B lymphocytes (CD19 1%, CD3 95%) and a decreased CD4+ T-cell count with an inversion of the ratio of the CD4+/CD8+ cells (0.29, cutoff 1–2); there was also mild IgM hypogammaglobulinemia (36 mg/dl; normal value 50–300), with normal values for the rest of the immunoglobulins. At admission, treatment with dexamethasone was initiated; the patient became afebrile 48 h later and with significant improvement in dyspnea. She was discharged with a prolonged tapering corticosteroid regimen. Prior to discharge, on day +36 of illness, a new RT-PCR was performed in plasma, again detecting the gene N (Ct 42.5). Five days after discharge, the patient again presented fever and dyspnea on slight efforts and was readmitted; low-flow oxygen supplementation was administered. Blood tests showed elevation of CRP (46 mg/L, cutoff <5), ferritin (564 ng/ml, cutoff <290), and IL-6 (52 pg/ml, cutoff <7). Remdesivir at standard doses was started, and one infusion of hyperimmune convalescent plasma was administered. In the following days, the patient presented a favorable clinical evolution, becoming afebrile 24 h after the administration of both treatments, with progressive improvement in dyspnea and oxygen saturation, staying above 95% without the need for oxygen supplies. After 8 days of treatment with remdesivir, on day +49 of illness, a SARS-CoV-2 RT-PCR was performed again in blood, still detecting gene N (Ct value similar to the previous one). Given the excellent status, the patient was discharged. A week after, on day +58 of illness, a new SARS-CoV-2 RT-PCR was performed in plasma, which was negative. She completed 2 months with prednisone; a new CT scan showed a resolution of bilateral infiltrates (**Figure 3B**). Since then, the patient has been well without recurrence of symptoms. Two months later, she became able to resume maintenance treatment with rituximab every 2 months for the lymphoproliferative syndrome. Nowadays, the patient has been able to restart her active lifestyle. We summarize the treatments received, clinical manifestations, and diagnostic tests in **Figure 4**.

## Discussion

In this report, we described two patients with B-cell lymphoma receiving rituximab who were vaccinated against SARS-CoV-2. They developed COVID-19 with protracted and recurrent courses of symptoms with persistent RNA detection in serum. Both were treated with remdesivir and convalescent plasma, besides immunomodulatory drugs.

Anti-CD20 drugs, such as rituximab, not only cause a malignant B-lymphocyte depletion but also deplete normal B cells and consequently induce a severe humoral immunity impairment (9). The association between increased risk of infections and rituximab therapy is well-established. In the PRIMA study, which assessed the potential benefit of 2 years of rituximab maintenance after first-line treatment in patients with follicular lymphoma receiving rituximab in addition to the chemotherapy regimen, infections were more frequent in patients receiving rituximab (10). Follicular non-Hodgkin lymphoma is a heterogeneous disease with a variable clinical course. It is considered a chronic disease for which conventional therapy is not curative, and most of these patients will finally develop progressive disease. The best predictor for tumor aggressiveness is the duration of remission. Therefore, anti-CD20 antibody-based maintenance therapy is indicated to achieve a maximum duration of the response. Using maintenance therapy during the COVID-19 pandemic was controversial. Some expert societies recommended sparing anti-CD20 antibody-based maintenance therapy for follicular lymphoma whenever possible, to avoid long-standing immunosuppression, or at least, an individual evaluation according to the local transmission of SARS-CoV-2^2^. In the first case, the patient had a high risk in the FLIPI index at the time of diagnosis, and after initial treatment, he reached a partial metabolic response. Following the current recommendations of expert societies of balancing individually the long-term outcome of anti-CD20 antibody-based maintenance against the risk of COVID-19 infection, we decided to continue rituximab. In the second case, despite our patient getting a complete response after the initial treatment, she was a non-older patient with a high burden of the disease at diagnosis (stage IV). For that reason, we decided to use maintenance therapy to obtain a long-term duration of the response as possible, avoiding the risk of recurrence.

About the possibility of postponement vaccination, a timeframe after ending rituximab to perform a sufficient response to the vaccine, it is well known that recovery of B-cell count after rituximab therapy usually occurs 6–9 months after the last dose, and normal levels are achieved after 9–12 months. The prolonged period of rituximab-induced depletion might impair humoral immune responsiveness to vaccination. Guidelines recommend waiting for at least 6 months after the last dose of rituximab for COVID-19 vaccination (11), but on the other hand, these patients are at very high risk of severe COVID-19 if infected, making the decisions in this context complex.

Some recent studies have shown that a high viral load of SARS-CoV-2 by RT-PCR in plasma seems to be associated with a worse COVID-19 prognosis (12, 13). Most of these studies did not identify which specific population had those outcomes but showed that hematological malignancies and COVID-19 are strongly associated with higher mortality and prolonged viral shedding in serum (14). In a cross-sectional study that included 41 patients, the authors found that viremic patients were more frequently affected by hematological malignancies (5 of 6 patients affected by hematological malignancies were viremic for SARS-CoV-2) (15). However, this association is based on a small-size population and needs to be corroborated. However, the fact that plasma viral load was not as high in subsequent episodes could be due to a partial immune response, although the technique for measuring it has not been validated in large-scale studies.

Until December 2021, we have attended ten patients with a history of lymphoma who were admitted to our hospital with severe COVID-19; 4 were under rituximab on maintenance therapy. The rest of these patients had received rituximab on different treatment regimens. Most patients had a severe and prolonged COVID-19, with persistent SARS-CoV-2 PCR positivity in their nasopharyngeal and respiratory samples and failed to develop anti-SARS-CoV-2 antibodies. A third of the patients died during their hospitalization and acute infection. Nevertheless, we only have confirmed the persistence of SARS-CoV-2 RNA in plasma by RT-PCR in these two cases presented. The first case did not develop anti-SARS-CoV-2 antibodies after 3 months of infection and failed also to do so even after the third dose of vaccination. The other one had a positive anti-S IgG and IgM but despite exposure to the virus never developed anti-N. About possible immunological hallmarks of chronic SARS-CoV-2 infections in these populations, Lee et al. suggest that combined depletion of B- and CD4+ T-cell counts induced by B-cell-directed therapies may contribute to developing a persistent symptomatic COVID-19 infection (16). Flow cytometry analysis in peripheral blood was performed in the two cases reported and revealed undetectable or very low B-cell counts. We also detected a decreased CD4+/CD8+ cell ratio in both patients.

In any case, we are not able to confirm if these two patients had a reinfection or reactivation. We could not perform a full sequence of the virus because only one sample showed Ct values below 30, and it was not available. The threshold ≤30 is considered the lower limit to obtain a reliable full genome sequence because SARS-CoV-2 has one of the RNA genomes with the most nucleotides. There is a well-known inverse relationship between viral load and the Ct value of conventional PCR tests; hence, it is internationally established that samples must have Ct ≤30 to perform this technique (17). However, we would like to point out several reasons for which we consider that reactivation is more probable: at that moment, the incidence of SARS-CoV-2 infection in our region has dropped from 580 to 47 cases per 100,000 inhabitants (18, 19); the patients did not report recent close contact with any positive person and have not been exposed in gatherings.

With regard to the treatments, we hypothesized that the antiviral effect of remdesivir and convalescent plasma contributed to viral clearance in serum and nasopharynx. In both cases, a long course of remdesivir of 10 days was carried out although a recent randomized open-label trial in severe COVID-19 patients not requiring ventilation did not demonstrate a significant difference between 5- and 10-day courses (20). However, the authors of this study specify that further evaluation must be conducted in immunocompromised patients as these patients were not representative. In the ACTT-1 study, only 8% of patients included in both arms had cancer; because no subgroup analysis was performed, and the type of cancer or chemotherapy received was not specified, the conclusions obtained may not be applicable to immunosuppressed patients (21).

In the past, convalescent plasma has been used in other viral infections like influenza and Middle East Respiratory Syndrome (22). However, the results of a recent meta-analysis on convalescent plasma suggest that it has no significant effect in reducing mortality among patients with severe COVID-19 (23); nevertheless, patients with B-cell depletion were typically excluded or underrepresented in the trials included in the meta-analysis. Due to technical difficulties, we were not able to measure the neutralizing capability of the convalescent plasma administered to both patients. At the time of occurrence of these two cases, anti-S monoclonal antibodies were not available; again, immunosuppressant patients were excluded in most trials evaluating these compounds (24). How the virus was finally cleared in both cases is unknown; we hypothesized that a combined treatment in cases were natural immunity is impaired would be the best option and preferably it would include monoclonal antibodies if available.

One of the limitations of our reports is our lack of capability to analyze a detailed T-cell adaptive immunity or whole-genome sequencing in different samples although we assume the same variant at each flare as the *Omicron* variant was not present in that moment and the *Delta* variant was predominant in Spain.

These cases illustrate the difficulty in treating COVID-19 infection in patients with anti-CD20 therapy in which knowledge about the performance of the infection was not well-known, but it was supposed that a long-term viral replication was due to the prolonged period of rituximab-induced B-cell depletion. The best way to deal with this situation in these patients is not well established, and it is mainly based on experts’ recommendations because this population is excluded or underrepresented in randomized clinical trials. We think the experience with these patients may provide some clues for the management of the infection in this scenario and open the door to new studies.

## Data Availability Statement

The raw data supporting the conclusions of this article will be made available by the authors, without undue reservation.

## Author Contributions

CM-C and LV-M wrote the first draft with supervision of IF-R and NP-C, respectively. JL-B did the analysis of RT-PCR and supervised microbiological aspects. LC-M, JR-B, and ZP-B corrected the latest version of the manuscript. All authors listed have made a substantial, direct, and intellectual contribution to the work and approved it for publication.

## Funding

This study was supported by Plan Nacional de I+D+i 2013‐2016, Instituto de Salud Carlos III, Subdirección General de Redes y Centros de Investigación Cooperativa, Ministerio de Ciencia, Innovación y Universidades, Spanish Network for Research in Infectious Diseases (REIPI RD16/0016/0001)—cofinanced by the European Development Regional Fund “A way to achieve Europe”, Operative Program Intelligence Growth 2014‐2020.

## Conflict of Interest

The authors declare that the research was conducted in the absence of any commercial or financial relationships that could be construed as a potential conflict of interest.

## Publisher’s Note

All claims expressed in this article are solely those of the authors and do not necessarily represent those of their affiliated organizations, or those of the publisher, the editors and the reviewers. Any product that may be evaluated in this article, or claim that may be made by its manufacturer, is not guaranteed or endorsed by the publisher.

## Footnotes

1
https://coronavirus.jhu.edu/map.html

2 ESMO, 2020. Cancer patient management during the COVID-19 pandemic. Available at: https://www.esmo.org/guidelines/cancer-patient-management-during-the-covid-19-pandemic (accessed December 27, 2021).
